# Machine Learning Models to Predict Kidney Stone Recurrence Using 24 Hour Urine Testing and Electronic Health Record-Derived Features

**DOI:** 10.21203/rs.3.rs-3107998/v1

**Published:** 2023-06-29

**Authors:** Patrick Doyle, Wu Gong, Ryan Hsi, Nicholas Kavoussi

**Affiliations:** Vanderbilt University; Vanderbilt University Medical Center; Vanderbilt University Medical Center; Vanderbilt University Medical Center

**Keywords:** machine learning, urolithiasis, recurrence, stone

## Abstract

**Objective:**

To assess the accuracy of machine learning models in predicting kidney stone recurrence using variables extracted from the electronic health record (EHR).

**Methods:**

We trained three separate machine learning (ML) models (least absolute shrinkage and selection operator regression [LASSO], random forest [RF], and gradient boosted decision tree [XGBoost] to predict 2-year and 5-year symptomatic kidney stone recurrence from electronic health-record (EHR) derived features and 24H urine data (n = 1231). ML models were compared to logistic regression [LR]. A manual, retrospective review was performed to evaluate for a symptomatic stone event, defined as pain, acute kidney injury or recurrent infections attributed to a kidney stone identified in the clinic or the emergency department, or for any stone requiring surgical treatment. We evaluated performance using area under the receiver operating curve (AUC-ROC) and identified important features for each model.

**Results:**

The 2- and 5- year symptomatic stone recurrence rates were 25% and 31%, respectively. The LASSO model performed best for symptomatic stone recurrence prediction (2-yr AUC: 0.62, 5-yr AUC: 0.63). Other models demonstrated modest overall performance at 2- and 5-years: LR (0.585, 0.618), RF (0.570, 0.608), and XGBoost (0.580, 0.621). Patient age was the only feature in the top 5 features of every model. Additionally, the LASSO model prioritized BMI and history of gout for prediction.

**Conclusions:**

Throughout our cohorts, ML models demonstrated comparable results to that of LR, with the LASSO model outperforming all other models. Further model testing should evaluate the utility of 24H urine features in model structure.

## Introduction

Kidney stone disease affects approximately 1 in 11 Americans during their lifetime and constitutes nearly $10 Billion in healthcare expenses annually [1–3]. After an index stone event, stone recurrence occurs in 35–50% of patients, with many requiring repeat interventions [4, 5]. Tools, such as the Recurrence of Kidney Stones (ROKS) nomogram, have been developed using clinical and demographic factors to predict stone recurrence [6]. Other features, however, may also predict stone events. For example, laboratory values or lithogenic medications have not been previously included in nomograms for prediction of stone recurrence. Moreover, 24-hour (24H) urine testing results, which can identify patients who may benefit from pharmacologic or dietary interventions for stone prevention, have not been included in prior models to predict stone recurrence [6, 7]. Robust models that leverage both EHR and 24H urine data have the potential to enable identification of patients who are at risk for symptomatic stone recurrence events.

Machine learning (ML) techniques offer a unique platform for integrating the complex clinical, demographic and laboratory data into a predictive model due to their ability to handle non-linear features with variable relationships [8]. Prior studies show feasibility of incorporating ML algorithms for clinical, patient evaluation [9, 10]. Additionally, these algorithms could be applied to 24-hour urine analysis to predict clinical outcomes and stone events. Despite previous attempts to predict symptomatic stone recurrence, no formal tool exists to predict stone recurrence using EHR and 24H urine data, further complicating interpretation [6, 7].

We sought to evaluate the feasibility of applying machine learning models for predicting symptomatic kidney stone recurrence from demographic, clinical and laboratory data. Additionally, we sought to assess which features were the strongest predictors of symptomatic stone recurrence in the model. To do this, we compared three machine learning models (least absolute shrinkage and selection operator [LASSO], random forest [RF], gradient boosted decision tree [XGBoost]) to logistic regression and evaluated prediction performance.

## Materials and Methods

### Patient Cohort

After local institutional review board approval, a retrospective review was performed of first-time symptomatic adult stone formers at our institution. All patients had completed 24-hour urine studies at a tertiary referral center between 2009 and 2021 (n = 1231). Demographic and clinical information was extracted in a semi-autonomous manner using research electronic data capture (REDCap) software from an institutionally maintained database [11–13]. Incident stone events were identified via International Classification of Disease (ICD) coding (Supplementary Table 1) using diagnosis of kidney stone, ureteral stone, hematuria, flank pain, and hydronephrosis. Outpatient, surgical, and emergency department visits associated with these codes were identified and then manually confirmed as related to the incident stone event. A specialized laboratory was used for all urine testing (Litholink Corporation, Chicago, IL) and stone composition analysis (Beck Laboratories, Greenwood, IN). Only one stone was analyzed per patient. If patients had history of multiple stones, analysis was only performed on the first known symptomatic stone.

### Stone recurrence

Stone recurrence was defined as an outpatient treatment, emergency department visit, or surgical intervention for kidney stone after the incident stone event. Recurrence events were identified via the same ICD coding as incident stones. All recurrences were manually validated. Recurrent stone events were only considered if they occurred over 90 days following the first stone event to account for repeat interventions for complications or staged surgeries. Additionally, asymptomatic or incidentally found stones were not counted as symptomatic recurrence. Symptomatic recurrence was evaluated within 2 years and 5 years following index stone event.

### Feature Selection

Clinical features were extracted based on ICD coding (Supplementary Table 2). Patient medications influential to stone formation were also recorded. Medications included prescription of an alkalinizing agent, allopurinol, or a thiazide diuretic were recorded (Supplementary Table 3).

The 24H urine study data was taken closest to the first stone event. Selected 24H urine features for ML models matched that of the minimum recommended features set by the American Urological Association [14]. These features include volume, urine pH, calcium, oxalate, citrate, uric acid, sodium, potassium, and creatinine. Stones compositions were categorized as calcium oxalate monohydrate, calcium oxalate dihydrate, calcium phosphate, uric acid, or other, which included dicalcium phosphate, struvite, ammonium hydrogen urate, carbonate apatite, cystine, and organic matter due to low sample count.

### Model Construction

For both the 2-year and 5-year recurrence prediction models, data were split chronologically into a 70% training cohort and 30% testing cohort based on index occurrence date such that the model operates predictively. Training and testing cohorts were then evaluated to ensure similarity.

Candidate features included patient demographic features, medical history, and urine and stone test. The features were determined by the investigators before modeling based on the literature, clinical relevance, and availability. Missing values on continuous variables were single imputed using predictive mean matching method. Categorical variables were converted to dummy variables before the modeling. The logistic regression (LR) model, the least absolute shrinkage and selection operator (LASSO) model, the Random Forest (RF) model, and the extreme gradient boosting (XGBoost) model were applied to make prediction. The LR, RF, and XGBoost models used all candidate features while LASSO had a variable selection process.

The models were built using the 70% training data, and the area under the curve (AUC) for the receiver operational characteristic curve was used to evaluate the model performance using the remaining 30% validation data. Variable importance was ranked by the Ward statistics in the LR model, the scaled coefficients in the LASSO model, the permutation importance in the RF model, and the Shapley Additive explanation (SHAP) value in the XGBoost model. All analyses were conducted using R version 4.1.3, the “mi” package was used for the single imputation, the “randomForest” package was used for the RF model, the “glmnet” package was use for the LASSO model, and the “xgboost” package for the XGBoost model [15–19].

### Performance Evaluation

The primary objective of this study was to develop machine learning models to predict symptomatic stone recurrence at 2- and 5-year intervals using demographic, clinical, stone, and urine data. Outcomes included the area under the receiver operating curve (AUC-ROC) for each of the 2- and 5-year predictive models. Secondary outcomes included the feature importance of each variable from the EHR-derived data used by the models for recurrence prediction. For LR, importance was ranked by relative feature importance. For LASSO, importance was determined based on the beta coefficients as determined by the model. For RF, importance as ranked by permutation, and for XGBoost, importance was ranked by SHAP value.

## Results

### Patient cohort

There were 1231 first-time symptomatic stone formers identified. There were 1104/1231 (90%) patients included in 2-year analyses with 585/1104 (53%) male, a mean age of 50 (SD = 15) years, and 278/1104 (25%) symptomatic recurrences. There were 875/1231 (71%) included in 5-year analyses with 470/875 (54%) male, a mean age of 50 (SD = 15) years, and 385/875 (44%) symptomatic recurrences. The remaining features used for model training are presented in Tables 1 and 2.

### Predictive Models

**Figure 1:**
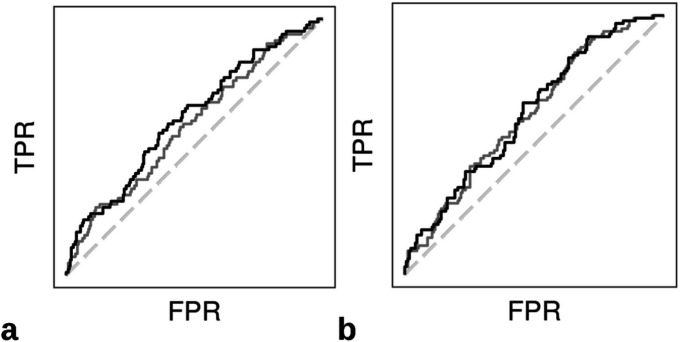
(a) 2-Year recurrence area under the receiver operating curves (AUC-ROC) for LASSO (black) and LR (grey) models. The AUC [95% CI] of LASSO was 0.617 [0.547, 0.687], and LR was 0.585 [0.514, 0.656]. (b) 5-Year recurrence AUC-ROC for LASSO (black) and LR (grey) models. The AUC [95% CI] of LASSO was 0.625 [0.557, 0.692], and LR was 0.618 [0.550, 0.686]. False positive rate (FPR), true positive rate (TPR), least absolute shrinkage and selection operator (LASSO), logistic regression (LR).

Evaluating 2-year symptomatic stone recurrence, the AUC for LR, LASSO, RF, and XGBoost models were 0.585, 0.617, 0.570 and 0.580, respectively (Table 3). The AUC for LR, LASSO, RF, and XGBoost models predicting 5-year symptomatic stone recurrence was 0.618, 0.625, 0.608, and 0.621, respectively. The LASSO model demonstrated superior performance among both 2- and 5-year recurrence models (Fig. 1).

### Predictive Features

Each model output individual rankings of features based on their importance in the model structure. Within the top 10 features of the 2- and 5-Year LASSO models, urine pH was the only feature derived from 24H Urine results (Supplementary Table 4). Type II diabetes mellitus and patient age ranked within the top 3 predictive features of both LASSO models. Patient age was the only feature found in the top 5 of every 2- and 5-year model tested. Within the 2-year recurrence models, none of the top 5 features for both LASSO and LR models were derived from 24H urines whereas 4/5 (80%) top features for both RF and XGBoost were derived from 24H urines. Urine pH ranked in the top 5 features of the 2- and 5-year RF and XGBoost models. In the 5-year recurrence models, again 0/5 (0%) top features for LASSO and LR models were from 24H urine studies.

### Comment

We demonstrate the feasibility of integrating 24H urine data into machine learning models for the prediction of kidney stone recurrence at 2 and 5 years following an index stone event. When comparing LASSO, RF, and XGBoost models to an LR model, the LASSO model performed superiorly with an AUC of 0.585 and 0.618 at 2 and 5 years, respectively. Patient age and a history of Type II diabetes mellitus were highly important features in the LASSO model, and patient age was highly important in all models.

The incorporation of complex, non-linear data to create predictive models is a recognized strength of ML models [20]. However, previous evaluations of symptomatic recurrence have focused on the use of traditional statistical approaches to form predictive models or identify risk factors of stone recurrence [4, 6, 21, 22]. Machine learning model performance in our study was similar to logistical regression and other linear models of prediction [23]. This demonstrates the workability of these models for predicting stone recurrence using large, non-linear datasets. We specifically found that the LASSO model outperformed logistic regression at recurrence prediction in our study. More robust datasets will enhance ML model performance.

Previously, Rule et al demonstrated 2- and 5-year prediction of stone recurrence after an index stone event at rates of 11% and 20%, respectively [6]. This study included 2239 first-time stone formers and identified younger age, male sex, and family history of stones as independent predictors of recurrence. At home 24H urine testing was not required for inclusion in this study. Our study population was higher risk as all required had 24H urine testing for inclusion. Thus, the identified 2- and 5-year recurrence rates (2-year: 25%, 5-year: 44%) of our study are higher than previously described [6].

Similar to prior studies, many features with known associations to stone recurrence were prioritized by the ML models [22, 24–28]. Specifically, age, diabetic status, urine pH and stone composition were among most important features utilized by all of the ML models. Thus, the machine learning models were able to prioritize features associated with known stone pathogenesis. Moreover, in the highest performing model (i.e, the LASSO model), the top features that predicted stone recurrence included age, type II diabetes, and BMI, which reflects known risk factors for recurrence.

This study and the incorporation of ML models has several limitations. Primarily, ML models require large, non-biased data sets to develop robust results [29]. The 2- and 5-year recurrence data sets containing 1104 and 875 patients, respectively, is below that which is considered a large ML dataset. Ensuring training and testing data have correctly assigned outcomes of recurrence is additionally important for model performance. This retrospective study could not account for patients who had symptomatic recurrence and did not present to our institution for treatment. Furthermore, post-treatment imaging was not required after index stone event to ensure stone free status prior to the tracking for stone recurrence. A 90-day wait period was enforced following index stone event to reduce false positives associated with index stone event. Lastly, the use of a single 24H urine to predict chronic stone formation lends results to the variability of 24H urine studies over time [14].

## Conclusion

ML models can analyze non-linear medical data, such as EHR-derived and 24H urine data, to develop predictive models of symptomatic kidney stone recurrence for first-time stone formers. Future studies are needed to build robust ML models that can more accurately predict symptomatic recurrence and guide the management of patients.

## Figures and Tables

**Table 1:** Cohort demographics and EHR features for patients with the 2- and 5-Year recurrence.

Patient Features	2-Year Recurrence n = 1104	5-Year Recurrence n = 875
Recurrence	278 (25)	385 (44.0)
Gender, Male	585 (53)	470 (54)
Age (years)*	50 (15)	50 (15)
BMI*	30 (7.8)	30 (7.8)
Hyperlipidemia	315 (29)	256 (29)
Bowel Disease	101 (9)	81 (9)
Osteoporosis, immobility, hyperparathyroidism	58 (5)	43 (5)
Epilepsy or migraines	48 (4)	40 (5)
Hypertension	602 (55)	480 (55)
Gout	48 (4)	37 (4)
Type 2 Diabetes Mellitus	247 (22)	191 (22)
Cystinuria	3 (0.3)	3 (0.3)
Coronary artery disease / MI	110 (10)	85 (10)
Cerebrovascular accident	31 (3)	25 (3)
Gastroesophageal reflux disease	396 (36)	328 (38)
Alkalinizing agent	92 (8)	63 (7)
Hydrochlorothiazide	72 (7)	60 (7)
Allopurinol	43 (4)	32 (4)

Unless otherwise indicated, data represent number of patients and parenthesis represent percentage of cohort.

* Data represent mean value and parenthesis represent standard deviation

**Table 2:** 24H urine metrics and stone type for 2- and 5-year recurrence cohorts

24H urine and stone features	2-Year Recurrence n = 1104	5-Year Recurrence n = 875
Urine Component*		
Urine volume [L/d]	2.01 (0.88)	2.00 (0.88)
Urine calcium	218 (127)	219 (125)
Urine oxalate	38 (18)	38 (18)
Urine citrate	619 (434)	618 (406)
Urine pH	6.1 (0.55)	6.1 (0.55)
Urine uric acid [g/d]	0.64 (0.25)	0.64 (0.25)
Urine sodium [mmol/d]	182 (85.9)	181 (85.9)
Urine potassium [mmol/d]	58 (26)	58 (25)
Urine creatinine [mg/d]	1677 (614.3)	1661 (606.9)
Majority stone composition		
Calcium oxalate monohydrate	617 (56)	479 (55)
Calcium oxalate dihydrate	116 (11)	92 (11)
Calcium phosphate	205 (19)	163 (19)
Uric Acid	86 (8)	73 (8)
Other	75 (7)	64 (7)
No single type	5 (0.5)	4 (0.5)

Unless otherwise indicated, data represent number of patients and parenthesis represent percentage of cohort. Urine metrics selected are based on the AUA minimum recommended features. Urine data is recorded from samples closest to the time of index symptomatic stone event.

* Data represent mean value and parenthesis represent standard deviation.

**Table 3:** Area under the curve (AUC) for the five predictive models

Model	2-Year AUC (95% CI)	5-Year AUC (95% CI)
LR	0.585 (0.514, 0.656)	0.618 (0.550, 0.686)
LASSO	0.617 (0.547, 0.687)	0.625 (0.557, 0.692)
RF	0.570 (0.498, 0.641)	0.608 (0.539, 0.677)
XGBoost	0.580 (0.505, 0.654)	0.621 (0.552, 0.690)

All AUCs were calculated using the 30% validation data that were not used in the predictive model building. All values in parenthesis denote upper and lower bounds of the 95% confidence interval. Least absolute shrinkage and selection operator (LASSO), random forest (RF), gradient boosted decision tree (XGBoost), and logistic regression (Lr).

## Data Availability

The authors confirm that the data supporting the findings of this study are available within the article and its supplementary materials.
